# Long QT syndrome in South Africa: the results of comprehensive genetic screening

**DOI:** 10.5830/CVJA-2013-032

**Published:** 2013-08

**Authors:** Paula L Hedley, Glenda A Durrheim, Firzana Hendricks, Valerie A Corfield, Cathrine Jespersgaard, Birgitte Støvring, Tam T Pham, Michael Christiansen, Althea Goosen, Paul A Brink

**Affiliations:** US/MRC Centre for Molecular and Cellular Biology, Department of Biomedical Sciences, Faculty of Medicine and Health Sciences, University of Stellenbosch, South Africa; Department of Clinical Biochemistry, Immunology and Genetics, Statens Serum Institut, Denmark; US/MRC Centre for Molecular and Cellular Biology, Department of Biomedical Sciences, Faculty of Medicine and Health Sciences, University of Stellenbosch, South Africa; US/MRC Centre for Molecular and Cellular Biology, Department of Biomedical Sciences, Faculty of Medicine and Health Sciences, University of Stellenbosch, South Africa; US/MRC Centre for Molecular and Cellular Biology, Department of Biomedical Sciences, Faculty of Medicine and Health Sciences, University of Stellenbosch, South Africa; Department of Clinical Biochemistry, Immunology and Genetics, Statens Serum Institut, Denmark; Department of Clinical Biochemistry, Immunology and Genetics, Statens Serum Institut, Denmark; Department of Clinical Biochemistry, Immunology and Genetics, Statens Serum Institut, Denmark; Department of Clinical Biochemistry, Immunology and Genetics, Statens Serum Institut, Denmark; Department of Internal Medicine, Faculty of Medicine and Health Sciences, University of Stellenbosch, South Africa; Department of Internal Medicine, Faculty of Medicine and Health Sciences, University of Stellenbosch, South Africa

**Keywords:** LQTS, mutation, ion-channels, sudden death, arrhythmia

## Abstract

**Abstract:**

Congenital long QT syndrome (cLQTS) is a genetic disorder predisposing to ventricular arrhythmia, syncope and sudden death. Over 700 different cLQTS-causing mutations in 13 genes are known. The genetic spectrum of LQTS in 44 South African cLQTS patients (23 known to carry the South African founder mutation p.A341V in *KCNQ1*) was established by screening for mutations in the coding regions of *KCNQ1*, *KCNH2*, *KCNE1*, *KCNE2* and *SCN5A*, the most frequently implicated cLQTS-causing genes (five-gene screening). Fourteen disease-causing mutations were identified, eight (including the founder mutation) in *KCNQ1*, five in *KCNH2* and one in *KCNE1*. Two mutations were novel. Two double heterozygotes were found among the 23 families (8.5%) carrying the founder mutation. In conclusion, cLQTS in South Africa reflects both a strong founder effect and a genetic spectrum similar to that seen in other populations. Consequently, five-gene screening should be offered as a standard screening option, as is the case internationally. This will disclose compound and double heterozygotes. Fivegene screening will most likely be even more informative in other South African sub-populations with a greater genetic diversity.

## Abstract

The congenital long QT syndrome (cLQTS)^1^ is an inherited disorder characterised by prolongation of the QT interval on a surface electrocardiogram (ECG) and an increased risk for life-threatening ventricular arrhythmias,^2^ particularly ventricular tachycardia of the torsades de pointes type. cLQTS provides the archetypical monogenic disorder for studying the genetic basis of inherited arrhythmia syndromes in that it is relatively common (prevalence of 1:2 500 to 1:5 000),^3^ and more than 700 disease-causing mutations have been identified in 13 genes encoding different cardiac ion channels or membrane adaptors.^4^,^5^ The genetic and allelic heterogeneity results in subtly different clinical phenotypes.^6^

Several clinical syndromes have been described: Romano-Ward syndrome (RWS), an autosomal dominant form of cLQTS, which presents clinically with a pure cardiac phenotype; Jervell and Lange-Nielsen syndrome (JLNS), an autosomal recessive form of LQTS which is associated with congenital deafness;^2^ Andersen-Tawil syndrome (ATS), which is an autosomal dominant multisystem disorder where cLQTS is variably present; and Timothy syndrome (TS), which is characterised by severe cLQTS as well as cardiac and somatic malformations.

cLQTS is associated with loss-of-function mutations in genes encoding repolarising K^+^ ion channels, their subunits and certain interacting proteins, i.e. *KCNQ1* encoding Kv7.1 (LQT1),^6^ *KCNH2* encoding Kv11.1 (LQT2),^7^ *ANK2* encoding Ankyrin B (LQT4),^8^ *KCNE1* encoding MinK (LQT5),^9^,^10^ *KCNE2* encoding MiRP1 (LQT6),^11^ *KCNJ2* encoding Kir2.1 (LQT7),^12^ *CAV3* encoding M-Caveolin (LQT9),^13^ *SCN4B* encoding Navβ3 (LQT10),^14^ *AKAP9* encoding Yotiao (LQT11),^15^ *SNTA1* encoding α1-Sytophin (LQT12)^16^ and *KCNJ5* encoding Kir3.4 (LQT13).^17^ Furthermore, gain-of-function mutations in genes encoding depolarising Na^+^ and Ca2^+^ ion channels have also been associated with LQTS, i.e. *SCN5A* (LQT3)^18^ and *CACNA1C* (LQT8).^19^

The ion channel defects result in either decreased K^+^ efflux or increased Na^+^ or Ca^2+^ influx over the cardiomyocyte plasma membrane, leading to reduced repolarisation and increased frequency of after-depolarisations (ADs) and prolonged refractory period. The latter predisposes to re-entrant ventricular arrhythmia.^4^ Adrenergic stimulation, which increases the frequency of ADs, may precipitate fatal arrhythmia in asymptomatic mutation carriers.^1^,^4^ Drugs, e.g. amiodarone, cisapride, sotalol and haloperidol, which reduce the repolarisation reserve, may cause drug-related LQTS (dLQTS) or, likewise, precipitate arrhythmia in mutation carriers.^1^,^4^

Previously, de Jager *et al.* and Brink *et al.* reported on the Kv7.1 (encoded by *KCNQ1*) founder mutation, p.A341V, identified in 23 Afrikaner families.^20^,^21^ Little is known of the occurrence of other cLQTS-associated mutations in South Africa.

In order to describe the spectrum of mutations causing cLQTS in South Africa, we screened 44 apparently unrelated South African cLQTS probands, including the 23 probands of the families that are carriers of the founder p.A341V mutation, for mutations in the five most frequently implicated cLQTS genes, *KCNQ1, KCNH2, SCN5A, KCNE1* and *KCNE2*, by multiplex capillary electrophoresis single-strand conformation polymorphism (multi-CE-SSCP). A sub-group was further analysed by the direct sequencing of these genes.

## Methods

Forty-four LQTS probands (23 known founder mutation carriers and 21 unrelated patients shown not to carry the founder mutation) [77% female; mean (SD) age at first event 10 (6) years, were ascertained to have cLQTS, mean (SD) QTc 501 (59) ms]. See Table 1 for clinical and demographic information. The diagnosis was based on the 1993 standard diagnostic criteria^22^ and included a physical examination, a standard 12-lead ECG and a personal interview. All probands were of the RWS type. All patients provided written informed consent (parental in the case of minors). The study was approved by the Health Research Ethics Committee of the University of Stellenbosch.

**Table 1:** Demographic And Clinical Characteristics Of 44 Index Patients

*Cohort*	*% female*	*QTc mean (SD)*	*Age at first event mean (SD)*
Total	77	501 (59)	10 (6)
A341V carriers	81	513 (48)	8 (4)
Other mutation carriers	73	507 (69)	11 (5)
No mutation detected	75	452 (35)	17 (7)

## Molecular genetic procedures

DNA was isolated from peripheral blood using a commercially available procedure (Qiagen GmBH, Germany). Primers were designed to amplify all the exons of the five LQTS-causing genes under investigation, including the intron/exon boundaries. The primers were synthesised by the Synthetic DNA Laboratory (University of Cape Town, Cape Town, South Africa) and Applied Biosystems (Copenhagen, Denmark).

PCR amplification was performed by published protocol for *SCN5A*.23 Primer sequences and PCR conditions for *KCNQ1*, *KCNH2*, *KCNE1* and *KCNE2* are available upon request (phy@ssi.dk). Primers used for generating PCR products for sequencing were tagged with M13 sequence. PCR products were qualitatively assessed on 2% agarose gels by standard procedures prior to multi-CE-SSCP and direct sequencing.

## Multi-CE-SSCP

PCR primers were labelled at their 5′ end with one of the following fluorophors: 6FAM^TM^, VIC®, NED^TM^ or PET^TM^ (Applied Biosystems, Foster City, California, USA). The labelled PCR amplicons were mixed and diluted according to published protocol^23^ for *SCN5A*. Amplicons of *KCNQ1, KCNH2, KCNE1* and *KCNE2* were mixed and diluted in a similar manner; the specifics of these mixtures are available upon request.

Multi-CE-SSCP was performed on an ABI Prism^TM^ 3100 Genetic Analyser (PE Applied Biosystems, Foster City, California, USA) with GeneScan polymer (Applied Biosystems, Foster City, California, USA) at 18°C and 30°C. PCR amplicons were sequenced to identify the variants responsible for alterations in the electrophoretic mobility detected by multi-CE-SSCP analysis.

## Direct sequencing

A sub-group of 32 probands was additionally analysed by direct DNA sequencing. The probands of the founder families were included to assess the frequency of compound heterozygozity in our cohort. PCR products were purified by exonuclease 1 and shrimp alkaline phosphatase reaction.

Sequencing of both strands was performed using BigDye^®^ Terminator v1.1 (Applied Biosystems, UK) and M13 primers (sense *5′-CAGTTCTCACAGGAGCCACA-3′*) and (antisense *5′-AGGTGAACTGGAACCACAGG-3′*) (Taq Copenhagen, Denmark). Sequences were analysed with the ABI 3730 DNA analyser (Applied Biosystem, UK).

## Bio-informatics

Nucleotide sequences were aligned to GenBank (http://www.ncbi.nlm.nih.gov/Entrez) reference sequences *KCNQ1* (NM_000218.2/NP_000209.2), *KCNH2* (NM_000238.2/NP_000229.1), SCN5A (NM_000335.2/NP_000326.2), *KCNE1* (NM_000219.2/NP_000210.2) and *KCNE2* (NM_172201.1/NP_75195.1). Multiple sequence alignments of multiple species were performed with Clustal W (version 1.82) (http://www.ebi.ac.uk/clustalw/#). SNPs were compared to the NCBI dbSNP database (http://www.ncbi.nlm.nih.gov/projects/SNP/) and the NHLBI Exome Sequencing Project Exome Variant Server (EVS) (http://evs.gs.washington.edu/EVS/). These data were used as *in silico* control.

## Criteria for disease association

A genetic variant was considered a disease-causing mutation if it resulted in an amino acid substitution, interfered with a splice site or had previously been shown to be associated with cLQTS and was not found in *in silico* controls and had not previously been described as a polymorphism. Sequence variants also found in controls were considered polymorphisms and are not reported here.

## Results

We identified 14 different disease-causing mutations in 34 of the 44 probands tested (Table 2). Three mutations were identified among the 23 founder probands; all harboured the South African founder mutation KCNQ1:p.A341V, while two probands (8.5%) harboured an additional mutation in a second cLQTS-causing gene, which may contribute to disease. These are cases of double heterozygosity. Furthermore, 11 mutations were identified in 13 non-founder probands.

**Table 2:** Disease-Causing Mutations Identified In The South African LQTS Probands As Well As An Indication Of Which Platform Identified The Variant

	*Mutations*		*Mutation detection technique*					
*Gene/protein*	*Coding substitution*	*Protein consequence*	*SSCP*	*sequencing*	*total*	*Frequency in in silico controls*	*Av QTc/additional info*	*References*
KCNQ1/Kv7.1	c.760G>A	p.V254M	1	ns	1	0	500 ms/ICD	4–6, 25–28, 33, 45, 55–58
	c.944A>G	p.Y315C	0	1	1	0	550 ms	4, 5, 25, 29, 33, 57, 59–62
	c.944A>C	p.Y315S	1	ns	1	0	512 ms	4, 26, 31, 57
	c.1022C>A	p.A341E	1	ns	1	0	502 ms/CA at 51 years	4, 5, 27, 32, 45, 57, 59, 63, 64
	c.1022C>T	p.A341V	23	23	23	0	513 ms/BB	4–6, 20, 21, 25–27, 45, 65–75
	c.1024C>T	p.L342F	1	1	1	0	527 ms	4, 5, 24, 26, 76, 77
	c.1031C>T	p.A344V	2	2	3^#^	0	557 ms	4, 5, 25, 26, 33, 34, 57, 68
	c.1760C>T	p.T587M	1	ns	1	0	400 ms	4, 5, 24, 35–37, 59, 78–80
	c.208C>A	p.R100W	1	1	1	0	437 ms	5
KCNH2/Kv11.1	c.917-3T>C	defective splicing/protein degradation	0	1	1	0	450 ms	This study
	c.982C>T*	p.R328C*	0	1	1	0	654 ms*	4, 5, 25, 30, 39, 40, 47, 48, 81–83
	c.1714G>A	p.G572S	1	1	1	0	402 ms/BB	4, 5, 24, 25, 39–44
	c.1882C>G	p.F627L	1	ns	1	0	462 ms	4, 45, 46
KCNE1/MinK	c.273C>A*	p.D91E*	1	1	1	0 (EA); 0.0002 (AA)	533 ms*	This study

*Variants that co-occur with Kv7.1:p.A341V; ^#^One of the probands in which p.A344V had been identified previously by SSCP was not available for sequencing. ns: not screened by sequencing; EA: 8 600 European-Americans screened by exome sequencing; AA: 4 406 African-Americans screened by exome sequencing; ICD: implantable cardioverter defibrillator; CA: cardiac arrest. BB: beta-blockers.

The multi-CE-SSCP results corroborated standard gel-based PCR-SSCP results (not shown), however, in 32 cases where the DNA concentration was sufficient, direct DNA sequencing led to the identification of four disease-causing mutations (KCNQ1:p.Y315C; KCNQ1:p.A344V; KCNH2:c.917-3T>C and KCNH2:p.R328C), which were missed by multi-CE-SSCP. In the case of KCNQ1:pA344V, two cases had been detected by SSCP, but an additional case was identified by direct sequencing.

## Discussion

We have identified 14 mutations in 34 of 44 (77 %) South African probands screened; two (14%) of the mutations identified are novel. The disease-causing mutations of 33 probands were identified by multi-CE-SSCP (Table 2), which represents a detection rate of 75%. Additionally, applying direct DNA sequencing to a smaller cohort resulted in the detection of the disease-causing mutation in 30 of the 32 probands screened, which represents a total detection rate of 94%.

These results are comparable to those reported in other screening studies.^22^,^24^,^25^ Therefore, in comparison to just screening for the KCNQ1:p.A341V founder mutation in our cLQTS probands (which had a detection rate of 52%), the addition of other genes and the application of direct DNA sequencing for mutation screening resulted in manifest improvements in diagnostic efficiency.

Within the founder families, represented by 23 probands, two probands (8.5 %) were double heterozygotes. One case carried KCNQ1:p.A341V and KCNH2:p.R328C while the other carried KCNQ1:p.A341V and KCNE1:p.D91E. This is an important finding as one of the purposes of genetic screening in cLQTS probands is the identification of asymptomatic mutation carriers in family members (cascade screening) in order to clinically assess the need for prophylaxis.

In theory, half of the mutation carriers within a cLQTS family may be missed if only one of the mutations in a compound heterozygote or double heterozygote is identified through selective screening for only one mutation or the selective screening of only one of the involved genes. As the clinical presentation may be sudden death in cLQTS mutation carriers, this is a serious clinical problem. Our results demonstrate that the detection of compound and double heterozygotes is also an important consideration in clinical handling of cLQTS cases in South Africa.

## KCNQ1 mutations

The common South African founder mutation (p.A341V) was identified in 23 probands.^20^,^21^ p.V254M was first identified in a large LQT1-kindred^6^ and later associated with sudden death before 40 years of age.^26^ This mutation was found to exert a dominant negative effect on native IKs.^27^ Assessment of betablocker therapy was performed on a cLQTS family carrying p.V254M; treatment was determined to be effective and safe.28

p.Y315C has been shown to be a dominant negative mutation, which, while the protein was normally trafficked to the cell surface, produced no measurable current.^29^ This mutation has been shown to be associated with cisapride-induced QT prolongation.^30^

p.Y315S was first identified in a French LQTS family.^26^ Jongbloed *et al.* (1999) identified this mutation as a *de novo* cause of cLQTS, which appeared to be triggered by both physical and emotional stress.^31^ In both cases the mutation was associated with an onset of symptoms before 10 years of age.

p.L342F was previously reported by Donger *et al*. (1997) where it was identified in a single case; none of the other family members carrying the mutation presented with symptoms of LQTS.^26^ However, the proband had experienced onset of symptoms before the age of 10 years.

p.A341E was identified in the first double heterozygote-carrying cLQTS family identified in a French cLQTS cohort,^32^ where carriers of both mutations (KCNQ1:p.A341E and KCNH2:c.2592+1G>A) were reported to be severely symptomatic, with both stress- and rest-induced symptoms initiating in early childhood. The mutation was found to exert a dominant negative effect on native IKs.^27^

The p.A344V mutation was first identified as a *forme fruste* cause of LQTS.^26^ Later, Choi *et al*. (2004) discovered this mutation in a cohort of patients, which had suffered swimming-triggered arrhythmias.^33^ In addition to shifting the voltage dependence of the IKs channel activation, the p.A344V mutation increased the sensitivity of the channel for bupivacaine (a local anaesthetic).^34^ KCNQ1:p.T587M was found to result in haplo-insufficiency as a consequence of being transport deficient.^35^,^36^ Furushima *et al*. (2010) identified a case of foetal atrioventricular block and unmasked maternal QT prolongation in the postpartum period in a mother and baby carrying this mutation.^37^

## KCNH2 mutations

KCNH2:c.917-3T>C has not been previously reported; however, splice-site analysis suggests that the tentative consequence of this variant is disruption the splice acceptor of exon 5. Such a splice-site disruption would be expected to cause aberrant mRNA splicing and result in either the synthesis of a truncated protein or, more likely, haplo-insufficiency. In both cases, the result is likely to be a reduced amount of Kv11.1 protein and therefore reduced repolarisation capacity.

KCNH2:p.R100W was identified in two of 2 500 patients reported by Kapplinger *et al*. in 2009, however, no specific clinical information is provided.^5^ A mutation at residue 100 (p.R100G) has been reported in a 41-year-old French woman who had suffered an aborted cardiac arrest,^38^ which may indicate that the R100 residue has a particular functional relevance.

The p.G572S mutation has been reported in LQTS cohorts around the world^4^,^5^,^23^,^25^,^39^-^44^ but has not been identified in an exome sequencing project to date. Zhao *et al.* in 2009 determined that p.G572S causes a dominant negative trafficking defect.^44^ KCNH2:p.F627L was first identified by Splawski *et al.* in 2000 as part of his LQTS screen of 262 probands^45^ and later was reported to be the cause of LQTS with foetal onset of atrioventricular block and ventricular tachycardia.^46^

## Double heterozygotes

In addition to the KCNQ1:p.A341V mutation, we have confirmed the presence of the KCNE1:p.D91E variant in a single proband. The clinical significance of this variant is unclear. It is a very rare variant, identified in a single African-American individual in an exome analysis of 4 406 African-American individuals (http://evs.gs.washington.edu/EVS/) (date accessed July 2012).

A more extensive analysis of the phenotypes in the family, where the clinical effect of individual mutations can be assessed as the *KCNQ1* mutation is located on chromosome 11 and the *KCNE1*-encoded MinK variant is located on chromosome 2,14 is necessary to establish the clinical significance of the compound heterozygozity. Due to the rarity of the MinK variant and the known association between mutations in the N-terminus of MinK and adverse drug effects, we consider it a significant finding.

In a second proband, we identified both the KCNQ1:p.A341V mutation and KCNH2:p.R328C. The p.R328C variant was first reported by Chevalier *et al.* (2001) in an acquired LQTS cohort.^47^ Grunnet *et al.* (2005) described a cLQTS patient harbouring double mutations (KCNQ1:pR591H; KCNH2:p.R328C). They determined that p.R328C did not produce a functional phenotype and that KCNQ1:p.R591H was sufficient to explain disease.^48^

Further functional assessment of KCNH2:p.R328C by Anderson *et al.* (2006) determined the Kv11.1 channels carrying p.R328C were normally trafficked to the membrane and conferred no functional phenotype.^39^ However, a subsequent report by Chevalier *et al.* (2007) suggested that p.R328C had a dominant negative effect on I_Kr_. Finally, Kapa *et al.* (2009) identified KCNH2:p.R328C in a control individual.^40^ The p.R328C variant is, in all likelihood, a rare polymorphism; however, we cannot exclude that it has a functional effect in the presence of certain drugs or conditions, e.g. hypokalaemia.^49^

Apart from the variants described above and reported in Table 2, a number of synonymous (i.e. not having any effect on the amino acid sequence) variants as well as variants that have been found with a relatively high frequency (i.e. > 1%) in other populations were found during the screening of the 44 probands (data not shown). Such polymorphisms are not disease causing, but they may modify the phenotype.^4^ These potential *forme fruste* mutations, i.e. mutations that do not appear to cause disease in isolation, should be noted.

Numerous non-synonymous polymorphisms have been reported to be associated with an effect in cardiac repolarisation currents.^50^ KCNE1:p.D85N has been implicated in drug-induced LQTS^30^ and KCNH2:p.R1047L has been reported to reduce I_Kr_ in a mammalian cell-based system.^51^

Occasionally, the presence of very rare variants in the normal population (e.g. the KCNE1: p.D91E mentioned above) that have not previously been associated with disease and have a population frequency less than 1:4 000 may raise questions about their possible role in disease causation. In such a case, family studies, which may not be possible in small families, and electrophysiological analysis, which is costly and time consuming, may be necessary to ascribe disease causation with certainty. Consequently, novel mutations where the disease association has not been established should be evaluated carefully and the uncertainty should be considered when deciding the appropriate management of the family, particularly as prophylactic treatment with beta-blockers or implantation of an ICD unit can have adverse effects.

To establish a genetic diagnosis in a cLQTS family is in most cases complicated, as described above, and is best done as a collaborative effort involving cardiologists, clinical geneticists and molecular geneticists. The findings reported here suggest that most South African cLQTS cases will be caused by mutations already described in other populations, as we only found two novel mutations. However, this probably reflects the fact that most of the patients included have a European ancestry and that to date no black South African LQTS patients have been encountered for enrolment in the study.

Furthermore, no other African-based research groups have reported the occurrence of LQTS patients within their black African populations.^52^ It must be expected, due to the large genetic diversity of African populations, as is evident from the Exome Sequencing Project (http://evs.gs.washington.edu/EVS/), that comprehensive genetic screening of at least five genes will be necessary to provide an adequate genetic diagnosis in these populations.

A limitation of this study was a failure to detect potential gene rearrangements, such as large duplications or deletions. Multi-CE-SSCP can only identify point mutations or small insertions and deletions in coding regions or at splice junctions. Koopman *et al.* detected a large duplication in the *KCNH2* gene, which they determined to be responsible for cLQTS in a Dutch family.^53^ No mutation has so far been identified in approximately 10–20% of cLQTS cases globally; gene rearrangements could account for a portion of these.

Furthermore, we only screened five of the 13 known LQTS-causing genes. However, the small sample size limits the possibility of identifying variants in genes that are rarely associated with cLQTS. These genes typically exhibit a prevalence of <1% of the LQTS population.^4^

## Conclusion

Genetic screening of five frequently implicated cLQTS causative genes in a predominantly white South African cLQTS cohort led to the identification of a disease-causing mutation in 77% of examined cases. The previously described founder mutation, p.A341V, is responsible for cLQTS in 52% of these probands, meaning that the extended screening increases the detection by 29% points. Furthermore, the frequency of double heterozygozity in South Africa is similar to the frequency seen in other populations.^54^ Therefore, 8.5% of the founder mutation-carrying families had members that were double heterozygotes.

These findings emphasise the importance of performing a comprehensive genetic screening when doing genetic work up, even in a population with a large founder effect. Despite the impressive diagnostic rate found here, it should be remembered that, using current techniques, it is not yet possible to establish a genetic diagnosis in many (23%) cLQTS families. In these cases, and wherever the clinical picture is complex, it is of paramount importance to realise that LQTS is a clinical condition requiring clinical management, irrespective of the genetic aetiology.
